# Supplementary material for: Persistence of symptoms and quality of life at 35 days after hospitalization for COVID-19 infection
Source: PLoS One. 2020 Dec 11;15(12):e0243882. doi: 10.1371/journal.pone.0243882 (PMC7732078; doi:10.1371/journal.pone.0243882)
Supplement: S1 File — (PDF) [file pone.0243882.s001.pdf]

Summary Data Demographics of Discharged COVID-19 Patients (n=183)

Variable	Level	Frequency(percentage)
Gender	Female	69(38.33)
Gender	Male	111(61.67)
Race	Black or African American	15(8.29)
Race	White	99(54.70)
Race	Asian	6(3.31)
Race	American Indian/Alaskan Native	2(1.10)
Race	Other	56(30.94)
Race	Unknown	3(1.66)
Ethnicity	Hispanic	54(30.00)
Ethnicity	Non-Hispanic	119(66.11)
Ethnicity	Unknown	7(3.89)
Other race	Arabic	1(4.76)
Other race	Cuban	1(4.76)
Other race	East Asian	1(4.76)
Other race	Filipino	7(33.33)
Other race	Hispanic	5(23.81)
Other race	Hispanic N	1(4.76)
Other race	Mixed	1(4.76)
Other race	Not Clear	1(4.76)
Other race	Not Listed	1(4.76)
Other race	Other Race	1(4.76)
Other race	Phillipino	1(4.76)
Hypertension	Unchecked	97(53.01)
Hypertension	Checked	86(46.99)
Diabetes	Unchecked	131(71.58)
Diabetes	Checked	52(28.42)
COPD	Unchecked	176(96.17)
COPD	Checked	7(3.83)
Asthma	Unchecked	164(89.62)
Asthma	Checked	19(10.38)
Cardiac issues	Unchecked	162(88.52)
Cardiac issues	Checked	21(11.48)
CRF/ESRD	Unchecked	175(95.63)

Variable	Level	Frequency(percentage)
CRF/ESRD	Checked	8(4.37)
CVD	Unchecked	176(96.17)
CVD	Checked	7(3.83)
Immuno-deficient disease	Unchecked	176(96.17)
Immuno-deficient disease	Checked	7(3.83)
Cancer	Unchecked	165(90.16)
Cancer	Checked	18(9.84)
None	Unchecked	143(78.14)
None	Checked	40(21.86)
Other comorbidity	Unchecked	108(59.02)
Other comorbidity	Checked	75(40.98)
Other Comorbidity	AFiib	1(1.33) cardiac arrhythmia
Other Comorbidity	Anxiety, depressi	1(1.33) psych
Other Comorbidity	Atrial fibrillati	1(1.33) cardiac arrythmia
Other Comorbidity	BPH, GERD	1(1.33) GERD
Other Comorbidity	Fatty liver, High	1(1.33) liver disease
Other Comorbidity	GERD	3(4.00) GERD
Other Comorbidity	GERD, Sleep Apnea	1(1.33) GERD, sleep apnea
Other Comorbidity	Gout	1(1.33) delete
Other Comorbidity	HIV	1(1.33) immunodeficiency
Other Comorbidity	HLD	4(5.33) hyperlipidemia
Other Comorbidity	HLD, Anxiety, IBS	1(1.33) hyperlipidemia, psych
Other Comorbidity	HLD, Depression	1(1.33) hyperlipidemia, psych
Other Comorbidity	Hepatitis B	1(1.33) liver disease
Other Comorbidity	Hyperlipidemia	2(2.67)hyperlipidema
Other Comorbidity	Hyperlipidemia, o	1(1.33) hyperlipidemia
Other Comorbidity	Hypothyroidism	3(4.00) delete

Variable	Level	Frequency(percentage)
Other Comorbidity	MS	1(1.33) immunodeficiency
Other Comorbidity	OSA, HLD	1(1.33) sleep apnea, hyperlipidemia
Other Comorbidity	PE, stem cell transplant	1(1.33) Thromboembolic disease, immunodeficiency
Other Comorbidity	PTSD, Depression	1(1.33)psych
Other Comorbidity	Pregnancy - 26 WG	1(1.33)pregnancy
Other Comorbidity	Pregnant	1(1.33)pregnancy
Other Comorbidity	Renal transplant	1(1.33)ESRD, immunodeficiency
Other Comorbidity	Rheumatoid arthritis	1(1.33)immunodeficiency (rheum)
Other Comorbidity	Schizophrenia	1(1.33)psych
Other Comorbidity	Sjogren's syndrome	1(1.33) immunodeficiency (rheum)
Other Comorbidity	alcoholic liver d	1(1.33) liver disease
Other Comorbidity	anxiety	1(1.33) psych
Other Comorbidity	arthritis, obesity	1(1.33) obesity
Other Comorbidity	chronic hepatitis	1(1.33) liver disease
Other Comorbidity	depression	2(2.67)psych
Other Comorbidity	dyslipidemia	1(1.33) hyperlipidemia
Other Comorbidity	elevated cholesterol	1(1.33) hyperlipidemia
Other Comorbidity	factor 9 deficiency	1(1.33) thromboembolic diease
Other Comorbidity	fatty liver	1(1.33) liver diseae
Other Comorbidity	gastric ulcer	1(1.33) skip
Other Comorbidity	hyperlipidemia	3(4.00) hyperlipidemia
Other Comorbidity	hyperlipidemia, O	1(1.33) hyperlipidemia
Other Comorbidity	hyperlipidemia, r	1(1.33) hyperlipidemia

Variable	Level	Frequency(percentage)
Other Comorbidity	hyperlipidemia, s	1(1.33) hyperlipidemia
Other Comorbidity	hypothyroidism	3(4.00) skip
Other Comorbidity	hypothyroidism(gr	1(1.33) skip
Other Comorbidity	hypothyroidism, u	1(1.33) skip
Other Comorbidity	liver disease	1(1.33) liver disease
Other Comorbidity	liver transplant,	1(1.33) liver disease, immunodeficiency
Other Comorbidity	lung cancer, rheumatism	1(1.33) cancer
Other Comorbidity	lupus, clotting d	1(1.33) thromboembolic disease
Other Comorbidity	obesity	1(1.33) obesity
Other Comorbidity	obesity (BMI 30.5	1(1.33) obesity
Other Comorbidity	obesity s/p obesity	1(1.33) obesity
Other Comorbidity	obstructive sleep	1(1.33) sleep apnea
Other Comorbidity	pregnant	1(1.33) pregnancy
Other Comorbidity	psoriasis	1(1.33) immunodeficiency (rheum)
Other Comorbidity	pulmonary embolism	1(1.33) thromboembolic disease
Other Comorbidity	rheumatoid arthritis	1(1.33) immunodeficiency (rheum)
Other Comorbidity	sarcoidosis	1(1.33) lung disease, other
Other Comorbidity	sciatic hernia	1(1.33) skip
Other Comorbidity	Sjogren’s syndrome	1(1.33) immunodeficiency (rheum)
Other Comorbidity	sleep apnea	1(1.33) sleep apnea
Other Comorbidity	sleep apnea, HLD	1(1.33) sleep apnea, hyperlipidemia
Other Comorbidity	sleep apnea, anxiety	1(1.33) sleep apnea, psych

Variable	Level	Frequency(percentage)
Other Comorbidity	sleep apnea, renal	1(1.33) sleep apnea
DVT	At admission	2(1.12)thromboem bolic disease
DVT	Developed during hospitalization for COVID-1	5(2.81) thromboembolic disease
DVT	Not applicable	171(96.07) thromboembolic disease
Cardiac CHF	Unchecked	178(97.27)cardiac HF
Cardiac CHF	Checked	5(2.73) cardiacHF
Cardiac MI	Unchecked	179(97.81) Cardiac CAD
Cardiac MI	Checked	4(2.19) Cardiac CAD
Cardiac CAD	Unchecked	175(95.63) Cardiac CAD
Cardiac cad	Checked	8(4.37) Cardiac CAD
Cardiac arrhythmia	Unchecked	178(97.27) Cardiac arrhythmia
Cardiac arrhythmia	Checked	5(2.73) Cardiac arrhythmia
Cardiomyopathy	Unchecked	182(99.45) Cardiac HF
Cardiomyopathy	Checked	1(0.55) Cardiac HF
Cardiac surgery sp	Unchecked?	181(98.91) Cardiac CAD?
Cardiac surgery sp	Checked	2(1.09) Cardiac CAD
Cardiac other	Unchecked	174(95.08) ??
Cardiac other	Checked	9(4.92) Cardiac NOS
Cardiac arrythmia	A	1(20.00) Cardiac arrhythmia
Cardiac arrythmia	a	2(40.00) Cardiac arrhythmia
Cardiac arrythmia	h	1(20.00) Cardiac arrhythmia
Cardiac arrythmia	p	1(20.00) Cardiac arrhythmia

Variable	Level	Frequency(percentage)
Other cardiac	LV thrombus	1(11.11) Thromboembolic disease
Other cardiac	Mitral valve prolapse, mitral regur	1(11.11) Cardiac VHD
Other cardiac	RBBB noted in admission note	1(11.11) Cardiac arrhythmia
Other cardiac	RVOT, s/p ablation	1(11.11) Cardiac CAD
Other cardiac	pacemaker, pulmonary hypertension	1(11.11) Cardiac arrhythmia
Other cardiac	pacemaker, stents	1(11.11) Cardiac arrhythmia, cardiac CAD
Other cardiac	s/p PCI (2011)	1(11.11) cardiac CAD
Other cardiac	valvular heart disease	1(11.11) cardiac VHD
Other cardiac	valvular heart disease, pulmonary h	1(11.11) cardiac VHD
Cancertypev2	All in remission on immunosuppressant	1(5.56) immunosuppressed
Cancertypev2	B-cell Lymphoma	1(5.56) immunosuppressed (cancer)
Cancertypev2	Bladder	1(5.56)
Cancertypev2	DLBCL, prostate	1(5.56)
Cancertypev2	Hairy cell leukemia	1(5.56) immunosuppressed (cancer)
Cancertypev2	MDS s/p BMT (11 yrs ago)	1(5.56) immunosuppressed (cancer)
Cancertypev2	Prostrate	1(5.56)
Cancertypev2	bladder , kidney , prostate	1(5.56)
Cancertypev2	breast cancer	2(11.11) cancer, solid
Cancertypev2	colorectal	1(5.56) cancer, solid
Cancertypev2	lung cancer	2(11.11) cancer, solid
Cancertypev2	multiple myeloma	2(11.11)immunosuppressed (cancer)

Variable	Level	Frequency(percentage)
Cancertypev2	prostate cancer	2(11.11) cancer, solid
Cancertypev2	thyroid	1(5.56) cancer, solid
All Entries Are Count(Percentages)		

Summary Data Discharge Symptoms of Discharged COVID-19 Patients (n=183)

Variable	Level	Frequency (percentage)
Discharge Symptoms	Yes	156 (85.25)
Discharge Symptoms	No	27 (14.75)
Symptom Discharge Shortness of Breath	Unchecked	89 (48.63)
Symptom Discharge Shortness of Breath	Checked	94 (51.37)
Symptom Discharge Cough	Unchecked	109 (59.56)
Symptom Discharge Cough	Checked	74 (40.44)
Symptom Discharge Fatigue	Unchecked	79 (43.17)
Symptom Discharge Fatigue	Checked	104 (56.83)
Symptom Discharge Muscle Pain	Unchecked	146 (79.78)
Symptom Discharge Muscle Pain	Checked	37 (20.22)
Symptom Discharge Joint Pain	Unchecked	165 (90.16)
Symptom Discharge Joint Pain	Checked	18 (9.84)
Symptom Discharge Headache	Unchecked	159 (86.89)
Symptom Discharge Headache	Checked	24 (13.11)
Symptom Discharge Lack of Taste	Unchecked	143 (78.14)
Symptom Discharge Lack of Taste	Checked	40 (21.86)
Symptom Discharge P Mouth Ulcer	Unchecked	180 (98.36)
Symptom Discharge P Mouth Ulcer	Checked	3 (1.64)
Symptoms Discharge Lack of Smell	Unchecked	155 (84.70)
Symptoms Discharge Lack of Smell	Checked	28 (15.30)
Symptoms Discharge Diarrhea	Unchecked	154 (84.15)
Symptoms Discharge Diarrhea	Checked	29 (15.85)
Symptoms Discharge Eye Irritation	Unchecked	170 (92.90)
Symptoms Discharge Eye Irritation	Checked	13 (7.10)

Variable	Level	Frequency (percentage)
Symptoms Discharge Phlegm	Unchecked	158 (86.34)
Symptoms Discharge Phlegm	Checked	25 (13.66)
Symptoms Discharge Fever	Unchecked	179 (97.81)
Symptoms Discharge Fever	Checked	4 (2.19)
Symptoms Discharge Confusion	Unchecked	168 (91.80)
Symptoms Discharge Confusion	Checked	15 (8.20)
Symptoms Discharge Other	Unchecked	149 (81.42)
Symptoms Discharge Other	Checked	34 (18.58)
Symptoms Discharge Not Answer	Unchecked	183 (100.00)
Other Symptoms Discharge	"throat ti	1 (3.03)
Other Symptoms Discharge	Anger	1 (3.03)
Other Symptoms Discharge	Constipation	1 (3.03)
Other Symptoms Discharge	GERD	1 (3.03)
Other Symptoms Discharge	Hair loss	1 (3.03)
Other Symptoms Discharge	Pleural pa	1 (3.03)
Other Symptoms Discharge	Stomach ac	1 (3.03)
Other Symptoms Discharge	Vasculitis	1 (3.03)
Other Symptoms Discharge	anxiety, d	1 (3.03)
Other Symptoms Discharge	burning chest	1 (3.03)
Other Symptoms Discharge	chest pain	1 (3.03)
Other Symptoms Discharge	chest tightness	2 (6.06)
Other Symptoms Discharge	difficulty	1 (3.03)
Other Symptoms Discharge	dizziness	3 (9.09)
Other Symptoms Discharge	dizzy - di	1 (3.03)
Other Symptoms Discharge	first week	1 (3.03)
Other Symptoms Discharge	had stroke	1 (3.03)
Other Symptoms Discharge	loss of ap	1 (3.03)
Other Symptoms Discharge	mild pain	1 (3.03)
Other Symptoms Discharge	minor pain	1 (3.03)
Other Symptoms Discharge	not s	1 (3.03)
Other Symptoms Discharge	numbness o	1 (3.03)
Other Symptoms Discharge	purple toe	1 (3.03)
Other Symptoms Discharge	rapid hear	1 (3.03)
Other Symptoms Discharge	smell heig	1 (3.03)
Other Symptoms Discharge	sore throat	1 (3.03)

Variable	Level	Frequency (percentage)
Other Symptoms Discharge	swollen le	1 (3.03)
Other Symptoms Discharge	trouble re	1 (3.03)
Other Symptoms Discharge	vertigo, d	1 (3.03)
Other Symptoms Discharge	vomiting	1 (3.03)
Discharged Home	Unchecked	13 (7.10)
Discharged Home	Checked	170 (92.90)
Discharged Home Care	Unchecked	176 (96.17)
Discharged Home Care	Checked	7 (3.83)
Discharged Sub Acute Facility	Unchecked	178 (97.27)
Discharged Sub Acute Facility	Checked	5 (2.73)
Discharged Require Oxygen	Unchecked	153 (83.61)
Discharged Require Oxygen	Checked	30 (16.39)
Discharged Not Answer	Unchecked	183 (100.00)

All entries are count(percentages)

Summary Data of Quality of Life of Discharged COVID-19 Patients (n=183)

**Living situation**	**Frequency, N (Percentages, %)**
Lives alone	22 (12.02)
Lives with family	147 (80.33)
Lives with roommates	8 (4.37)
Lives with boyfriend/girlfriend/fiancé	4 (2.20)
Other-unspecified	1 (0.55)
I do not want to answer this question	1 (0.55)
**Missing values: 0**	

**Do you have any other family member that got sick with COVID-19?**	**Frequency, N (Percentages, %)**
Yes	90 (52.02)
Was your family member hospitalized?	
Yes	22 (24.72)
No	67 (75.28)
No	81 (46.82)
I do not want to answer this question	2 (1.16)
**Missing values: 10**	

How is your family member doing now?	Frequency, N (Percentages, %)
Fully recovered / back to normal	67 (79.06)
Better, recovering (but not fully recovered)	10 (10.18)
Still experiencing symptoms	3 (3.53)
Still sick	2 (2.35)
Deceased	3 (3.53)
**Missing values: 5 (of the 90 that reported that someone got sick above)**	

Current employment status	Frequency, N (Percentages, %)
Employed	95 (52.20)
Unemployed	87 (47.80)
**Missing values: 1**	

Currently working (return to work)	Frequency, N (Percentages, %)
Employed	49 (29.88)
Unemployed	114 (69.51)
I do not want to answer this question	1 (0.61)
**Missing values: 1**	

	Level				
**Category**	**Poor**	**Fair**	**Good**	**Very good**	**Excellent**
**General Health**	2 (1.09)	35 (19.13)	70 (38.25)	57 (31.15)	19 (10.38)
**Quality of Life**	7 (3.87)	35 (19.34)	67 (37.02)	47 (25.97)	25 (13.81)
**Overall Physical Health**	4 (2.21)	45 (24.86)	62 (34.25)	57 (31.49)	13 (7.18)
**Overall Mental Health**	4 (2.19)	27 (14.75)	72 (39.34)	50 (27.32)	30 (16.39)
**Social Relationships**	9 (4.95)	38 (20.88)	63 (34.62)	49 (26.92)	23 (12.64)
**Social Activities, Roles**	8 (4.42)	49 (27.07)	54 (29.83)	49 (27.07)	21 (11.60)

	Level				
**Category**	**Not at all**	**A little**	**Moderately**	**Mostly**	**Completely**
**Daily Physical Activity**	1 (0.55)	24 (13.26)	45 (24.86)	51 (28.18)	60 (33.15)
	**Always**	**Often**	**Sometimes**	**Rarely**	**Never**
**Emotional problems**	0	26 (14.21)	64 (34.97)	39 (21.31)	54 (29.51)
	**Very Severe**	**Severe**	**Moderate**	**Mild**	**None**
**Fatigue**	1 (0.55)	14 (7.65)	60 (32.79)	57 (31.15)	51 (27.87)

	Level				
**Category**	**No difficulty**	**A little difficulty**	**Some difficulty**	**Much difficulty**	**I did not do this in the past 7 days**
**Dressing**	166 (90.71)	11 (6.01)	3 (1.64)	3 (1.64)	0
**Walking**	112 (61.54)	34 (18.68)	20 (10.99)	7 (3.85)	9 (4.95)
**20 stairs (2 flights) without stopping**	66 (36.07)	49 (26.78)	31 (16.94)	18 (9.84)	19 (10.38)
**Meal preparation**	128 (70.72)	20 (11.05)	8 (4.42)	2 (1.10)	23 (12.71)
**Wash dishes**	140 (77.35)	13 (7.18)	3 (1.66)	4 (2.21)	21 (11.60)
**Sweeping or mopping**	115 (63.89)	18 (10.00)	12 (6.67)	6 (3.33)	29 (16.11)
**Making the bed**	134 (74.03)	16 (8.84)	5 (2.76)	5 (2.76)	21 (11.60)
**Lifting something weighing 10-20 lbs**	92 (50.27)	37 (20.22)	23 (12.57)	10 (5.46)	21 (11.48)
**Carrying something weighing 10-20 lbs (about 4.5-9kg, like a large bag of groceries) from one room to another**	77 (42.08)	43 (23.50)	27 (14.75)	14 (7.65)	22 (12.02)
**Walking (faster than your usual speed) for ½ mile (almost 1 km) without stopping**	39 (21.31)	35 (19.13)	33 (18.03)	29 (15.85)	47 (25.68)

Univariable Analysis of Comorbidities vs General Health By Day 35 post discharge

Outcome	*Effect*	*Odds Ratio*	*95% CI*	*P-Value*	*Pr > Chi-Square*
General Health	General Health Very good, excellent: gender Female vs Male	0.808	(0.361,1.811)	0.6050	0.7888
General Health	General Health Good: gender Female vs Male	1.002	(0.444,2.259)	0.9969	0.7888
General Health	General Health Very good, excellent: AgeOlder Age>=60 vs Age	0.955	(0.432,2.112)	0.9086	0.7186
General Health	General Health Good: Age Older Age>=60 vs Age<60	1.240	(0.556,2.763)	0.5995	0.7186
General Health	General Health Very good, excellent: Obese Obese vs Not Obes	2.034	(0.912,4.539)	0.0828	0.0917
General Health	General Health Good: Obese Obese vs Not Obese	1.063	(0.472,2.395)	0.8822	0.0917
General Health	General Health Very good, excellent: Hypertension Checked vs	0.723	(0.328,1.594)	0.4212	0.3121
General Health	General Health Good: Hypertension Checked vs Unchecked	0.539	(0.241,1.206)	0.1326	0.3121
General Health	General Health Very good, excellent: Diabetes Checked vs Unc	0.627	(0.273,1.443)	0.2723	0.3334
General Health	General Health Good: Diabetes Checked vs Unchecked	0.527	(0.223,1.245)	0.1441	0.3334
General Health	General Health Very good, excellent: CardiacIssues Checked v	0.649	(0.191,2.203)	0.4885	0.7188
General Health	General Health Good: CardiacIssues Checked vs Unchecked	0.944	(0.292,3.055)	0.9238	0.7188
General Health	General Health Very good, excellent: None Checked vs Uncheck	1.488	(0.533,4.156)	0.4482	0.6281
General Health	General Health Good: None Checked vs Unchecked	1.656	(0.591,4.643)	0.3372	0.6281

Univariable Analysis of Comorbidities vs Quality of Life By Day 35 post discharge

Outcome	*Effect*	*Odds Ratio*	*95% CI*	*P-Value*	*Pr > Chi-Square*
Quality of Life	Quality of Life Very good, excellent: gender Female vs Male	0.516	(0.236,1.129)	0.0977	0.2353
Quality of Life	Quality of Life Good: gender Female vs Male	0.762	(0.349,1.660)	0.4934	0.2353
Quality of Life	Quality of Life Very good, excellent: AgeOlder Age>=60 vs Age	0.660	(0.305,1.427)	0.2908	0.2688
Quality of Life	Quality of Life Good: AgeOlder Age>=60 vs Age<60	1.133	(0.524,2.453)	0.7507	0.2688
Quality of Life	Quality of Life Very good, excellent: Obese Obese vs Not Obes	2.571	(1.155,5.726)	0.0207	0.0689
Quality of Life	Quality of Life Good: Obese Obese vs Not Obese	1.872	(0.838,4.182)	0.1263	0.0689
Quality of Life	Quality of Life Very good, excellent: Hypertension Checked vs	0.895	(0.418,1.916)	0.7747	0.9579
Quality of Life	Quality of Life Good: Hypertension Checked vs Unchecked	0.914	(0.423,1.978)	0.8200	0.9579
Quality of Life	Quality of Life Very good, excellent: Diabetes Checked vs Unc	0.690	(0.294,1.614)	0.3917	0.4576
Quality of Life	Quality of Life Good: Diabetes Checked vs Unchecked	1.091	(0.476,2.500)	0.8377	0.4576
Quality of Life	Quality of Life Very good, excellent: CardiacIssues Checked v	0.625	(0.209,1.868)	0.4001	0.4745
Quality of Life	Quality of Life Good: CardiacIssues Checked vs Unchecked	0.492	(0.153,1.580)	0.2333	0.4745
Quality of Life	Quality of Life Very good, excellent: None Checked vs Uncheck	1.545	(0.582,4.104)	0.3827	0.6726
Quality of Life	Quality of Life Good: None Checked vs Unchecked	1.442	(0.534,3.897)	0.4706	0.6726

Univariable analysis of Comorbidities vs Physical Healthy By Day 35 post discharge

Outcome	*Effect*	*Odds Ratio*	*95% CI*	*P-Value*	*Pr > Chi-Square*
Physical Health	Physical Health Very good, excellent: gender Female vs Male	0.417	(0.194,0.894)	0.0246	0.0721
Physical Health	Physical Health Good: gender Female vs Male	0.724	(0.339,1.543)	0.4025	0.0721
Physical Health	Physical Health Very good, excellent: AgeOlder Age>=60 vs Age	0.929	(0.447,1.929)	0.8435	0.6124
Physical Health	Physical Health Good: AgeOlder Age>=60 vs Age<60	0.704	(0.331,1.499)	0.3624	0.6124
Physical Health	Physical Health Very good, excellent: Obese Obese vs Not Obes	1.933	(0.912,4.096)	0.0853	0.2170
Physical Health	Physical Health Good: Obese Obese vs Not Obese	1.341	(0.625,2.879)	0.4512	0.2170
Physical Health	Physical Health Very good, excellent: Hypertension Checked vs	0.533	(0.254,1.121)	0.0972	0.0842
Physical Health	Physical Health Good: Hypertension Checked vs Unchecked	0.428	(0.199,0.921)	0.0300	0.0842
Physical Health	Physical Health Very good, excellent: Diabetes Checked vs Unc	0.308	(0.138,0.686)	0.0040	0.0055
Physical Health	Physical Health Good: Diabetes Checked vs Unchecked	0.330	(0.145,0.747)	0.0079	0.0055
Physical Health	Physical Health Very good, excellent: CardiacIssues Checked v	0.925	(0.299,2.856)	0.8918	0.9050
Physical Health	Physical Health Good: CardiacIssues Checked vs Unchecked	0.768	(0.231,2.548)	0.6660	0.9050
Physical Health	Physical Health Very good, excellent: None Checked vs Uncheck	2.822	(0.964,8.263)	0.0583	0.0636
Physical Health	Physical Health Good: None Checked vs Unchecked	3.600	(1.228,10.55)	0.0196	0.0636

Univariable analysis of Comorbidities vs Mental Healthy By Day 35 post discharge

Outcome	*Effect*	*Odds Ratio*	*95% CI*	*P-Value*	*Pr > Chi-Square*
Physical Health	Physical Health Very good, excellent: gender Female vs Male	0.374	(0.160,0.877)	0.0236	0.0759
Physical Health	Physical Health Good: gender Female vs Male	0.536	(0.229,1.258)	0.1520	0.0759
Physical Health	Physical Health Very good, excellent: AgeOlder Age>=60 vs Age	1.170	(0.501,2.732)	0.7162	0.4922
Physical Health	Physical Health Good: AgeOlder Age>=60 vs Age<60	1.583	(0.672,3.733)	0.2937	0.4922
Physical Health	Physical Health Very good, excellent: Obese Obese vs Not Obes	1.945	(0.832,4.548)	0.1247	0.2381
Physical Health	Physical Health Good: Obese Obese vs Not Obese	1.299	(0.549,3.072)	0.5513	0.2381
Physical Health	Physical Health Very good, excellent: Hypertension Checked vs	0.873	(0.380,2.003)	0.7481	0.8227
Physical Health	Physical Health Good: Hypertension Checked vs Unchecked	1.067	(0.459,2.477)	0.8806	0.8227
Physical Health	Physical Health Very good, excellent: Diabetes Checked vs Unc	0.895	(0.345,2.327)	0.8207	0.3093
Physical Health	Physical Health Good: Diabetes Checked vs Unchecked	1.529	(0.598,3.912)	0.3756	0.3093
Physical Health	Physical Health Very good, excellent: CardiacIssues Checked v	0.528	(0.171,1.634)	0.2678	0.2875

Outcome	Effect	Odds Ratio	95% CI	P-Value	Pr > Chi-Square
Physical Health	Physical Health Good: CardiacIssues Checked vs Unchecked	0.379	(0.112,1.285)	0.1193	0.2875
Physical Health	Physical Health Very good, excellent: None Checked vs Uncheck	1.042	(0.366,2.965)	0.9390	0.7086
Physical Health	Physical Health Good: None Checked vs Unchecked	1.389	(0.492,3.924)	0.5353	0.7086

Univariable analysis of Comorbidities vs Social Relationships By Day 35 post discharge

Outcome	Effect	Odds Ratio	95% CI	P-Value	Pr > Chi-Square
Social Relationships	gender Female vs Male	0.523	(0.278,0.986)	0.0451	0.0451
Social Relationships	AgeOlder Age>=60 vs Age	0.725	(0.398,1.323)	0.2953	0.2953
Social Relationships	Obese Obese vs Not Obes	1.035	(0.568,1.888)	0.9100	0.9100
Social Relationships	Hypertension Checked vs	0.962	(0.531,1.745)	0.8992	0.8992
Social Relationships	Diabetes Checked vs Unc	0.745	(0.382,1.455)	0.3890	0.3890
Social Relationships	CardiacIssues Checked v	0.738	(0.283,1.929)	0.5361	0.5361
Social Relationships	None Checked vs Uncheck	0.943	(0.456,1.951)	0.8747	0.8747
